# Supplementary material for: A Study of Vanadate Group Substitution into Nanosized Hydroxyapatite Doped with Eu3+ Ions as a Potential Tissue Replacement Material
Source: Nanomaterials (Basel). 2021 Dec 28;12(1):77. doi: 10.3390/nano12010077 (PMC8746586; doi:10.3390/nano12010077)
Supplement: Supplementary file 1 [file nanomaterials-12-00077-s001.zip › nanomaterials-1425957-supplementary.pdf]

nanomaterials

MDPI

Supplementary Materials

A Study of Vanadate Group Substitution into Nanosized Hydroxyapatite Doped with Eu^{3+} Ions as a Potential Tissue Replacement Material

Nicole Nowak* and Rafal Jakub Wiglusz*

Institute of Low Temperature and Structure Research, Polish Academy of Sciences, Okolna 2, 50-422 Wroclaw, Poland

* Correspondence: n.nowak@intibs.pl (N.N.); r.wiglusz@intibs.pl (R.J.W.); Tel.: +48-071-3954-159 (N.N. & R.J.W.)

Abstract: In this study, nanosized vanadate-substituted hydroxyapatites doped with 1 mol% and 2 mol% Eu^{3+} ions were obtained via the precipitation method. To evaluate the structure and morphology of the obtained compounds, the XRPD (X-ray powder diffraction) technique, Rietveld refinement, SEM-EDS (scanning electron microscopy-energy-dispersive spectrometry) and TEM (transmission electron microscopy) techniques as well as FTIR (Fourier transform infrared) spectroscopy were performed. Moreover, the chemical formula was confirmed using the ICP-OES (Inductively coupled plasma optical emission spectroscopy spectroscopy). The calculated average grain size for powders was in the range of 25 to 90 nm. The luminescence properties of vanadium-substituted hydroxyapatite were evaluated by recording emission spectra and excitation spectra as well as luminescence kinetics. The crucial step of this research was the evaluation of the biocompatibility of the synthesized nanomaterials. Therefore, the obtained compounds were tested toward sheep red blood cells and normal human dermal fibroblast to confirm the nontoxicity and biocompatibility of new nanosized Eu^{3+} ion-doped vanadate-hydroxyapatite. Moreover, the final step of the research allowed us to determine the time dependent ion release to the simulated body fluid environment. The study confirmed cytocompatibility of vanadium hydroxyapatite doped with Eu3+ ions.

Table S1. Unit cell parameters (a-c), crystal cell volume (V), as well as refined factor (R_w) for the $Ca_{10}(PO_4)_5(VO_4)_1(OH)_2$ (HAVp) and $Ca_{10}(PO_4)_{6-x}(VO_4)_x(OH)/Ca_2V_2O_7$ (HAVp/CaVO) nanocomposites doped with x mol% Eu^{3+} (where x = 0-2).

	**Cell parameters**										**Phase**		
	**Hydroxyapatite $Ca_5(PO_4)_3OH$**			**calcium pyrovanadate $Ca_2V_2O_7$**							**HAVp**	**CaVO**	**R_w**
**Sample**	**a (Å)**	**c (Å)**	**V (Å³)**	**a (Å)**	**b (Å)**	**c (Å)**	**α (°)**	**β (°)**	**γ (°)**	**V (Å³)**	**(%)**	**(%)**	**–**
**s. c.**	9.424(4)	6.879(4)	529.09(54)	6.6699(6)	6.9197(6)	7.0157(6)	86.39(0)	63.84(0)	83.67(0)	288.83(15)	–	–	–
1 mol% Eu^{3+}:$Ca_{10}(PO_4)_{6-x}(VO_4)_x(OH)/Ca_2V_2O_7$													
**HAVp**	9.466(2)	6.898(2)	535.32(53)								100.00		2.10
**x – 2**	9.484(9)	6.906(2)	538.06(57)	6.6305(8)	6.9364(9)	6.9848(8)	86.33(4)	64.21(8)	84.08(3)	287.66(94)	92.39	7.61	2.15
**x – 3**	9.509(8)	6.931(9)	542.90(73)	6.6920(4)	6.8877(8)	7.0312(9)	86.54(7)	63.75(3)	83.61(3)	288.85(35)	47.60	52.40	2.30
**x – 4**	9.523(4)	6.922(7)	543.73(86)	6.6666(4)	6.9201(1)	7.0158(4)	86.55(7)	63.85(7)	83.77(0)	288.81(27)	57.41	42.59	2.28
**x – 5**	9.519(9)	6.929(0)	543.83(35)	6.6684(6)	6.9220(4)	7.0182(1)	86.51(1)	63.86(5)	83.72(3)	289.06(15)	37.62	62.38	2.00
**CaVO**				6.6700(1)	6.9248(5)	7.0211(7)	86.45(5)	63.86(2)	83.68(7)	290.25(11)		100	2.01
2 mol% Eu^{3+}:$Ca_{10}(PO_4)_{6-x}(VO_4)_x(OH)/Ca_2V_2O_7$													
**HAVp**	9.465(9)	6.903(0)	536.35(45)								100		2.11
**x – 2**	9.488(8)	6.911(9)	538.95(27)	6.6702(0)	6.9194(4)	7.0155(4)	86.38(7)	63.84(2)	83.66(9)	288.82(29)	95.10	4.90	2.20
**x – 3**	9.512(1)	6.924(7)	542.60(58)	6.6631(4)	6.9050(2)	7.0054(2)	86.56(9)	63.89(2)	83.80(3)	287.70(76)	78.20	21.80	2.30
**x – 4**	9.531(7)	6.931(4)	545.37(14)	6.6763(1)	6.9248(2)	7.0262(7)	86.47(6)	63.83(7)	83.69(3)	289.76(26)	36.56	63.44	2.40

**x – 5**	9.489(4)	6.938(2)	541.07(19)	6.6721(5)	6.9238(1)	7.0192(7)	86.47(9)	63.84(4)	83.67(7)	289.26(06)	13.72	86.28	2.35
**CaVO**				6.6694(7)	6.9242(8)	7.0207(5)	86.45(7)	63.85(6)	83.67(7)	289.25(28)		100	2.03

s. c. – single crystal reference data, $Ca_5(PO_4)_3OH$ –26204-ICSD, $Ca_2V_2O7_4$ – 421266-ICSD

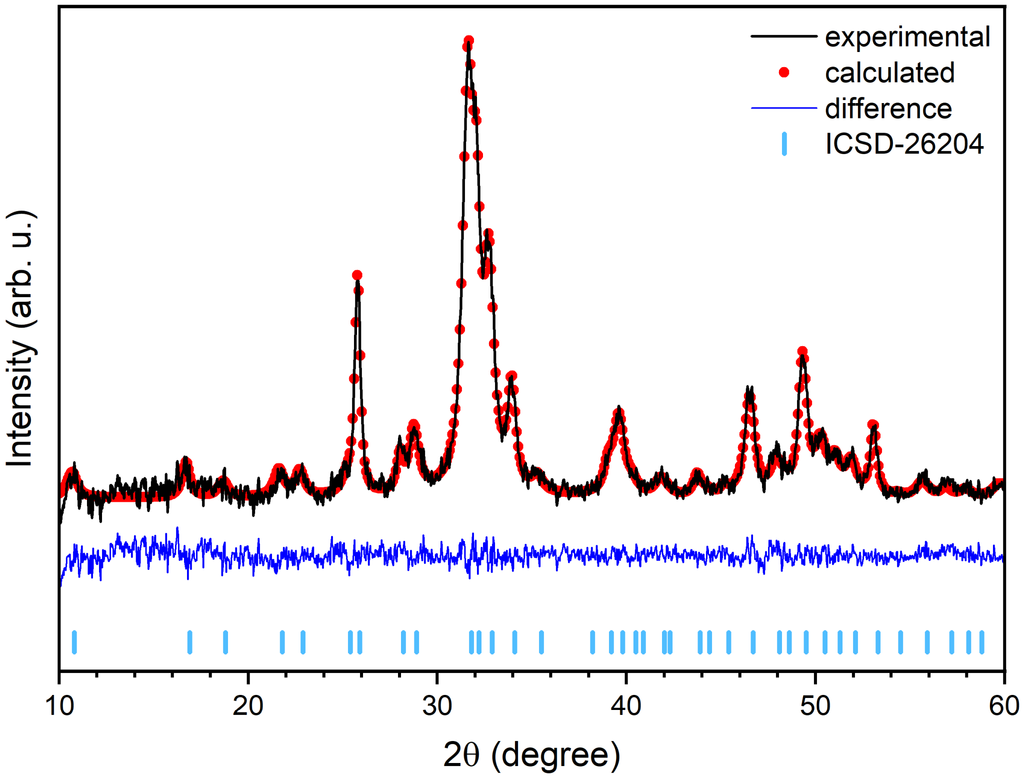

Figure S1. Representative results of the of the 1 mol% Eu^{3+} ion doped $Ca_{10}(PO_4)_5(VO_4)_1(OH)_2$, obtained at 600°C, Rietveld analysis (red – fitted diffraction; blue – differential pattern, column – reference phase peak position).
